# The spectrum of acute illness and mortality of children and adolescents presenting to emergency services in Sanghar district hospital, Pakistan: a prospective cohort study

**DOI:** 10.1136/bmjopen-2023-082255

**Published:** 2024-08-22

**Authors:** Fiona Muttalib, Zahid Ali Memon, Shah Muhammad, Asif Soomro, Samia Khan, Shazia Bano, Muhammad Jawwad, Sajid Soofi, Bettina Hansen, Neill KJ Adhikari, Zulfiqar Bhutta

**Affiliations:** 1 Department of Pediatrics, BC Children's Hospital, Vancouver, British Columbia, Canada; 2 Center of Excellence in Women & Child Health, The Aga Khan University, Karachi, Sindh, Pakistan; 3 Pediatrics & Child Health, Aga Khan University, Karachi, Pakistan; 4 University of Toronto, Toronto, Ontario, Canada; 5 Interdepartmental Division of Critical Care Medicine and Institute of Health Policy, Managament, and Evaluation, University of Toronto, Toronto, Ontario, Canada; 6 Department of Critical Care Medicine, Sunnybrook Health Sciences Centre, Toronto, Ontario, Canada; 7 Centre for Global Child Health, Hospital for Sick Children Research Institute, Toronto, Ontario, Canada

**Keywords:** Epidemiology, PAEDIATRICS, Paediatric A&E and ambulatory care

## Abstract

**Abstract:**

**Objective:**

To describe presenting diagnoses and rates and causes of death by age category and sex among children with acute illness brought to a district headquarter hospital in Pakistan.

**Design:**

Prospective cohort study.

**Setting:**

Sanghar district headquarter hospital, Sindh, Pakistan between December 2019 and April 2020 and August 2020 and December 2020.

**Participants:**

3850 children 0–14 years presenting with acute illness to the emergency and outpatient departments and 1286 children admitted to the inpatient department.

**Outcome measures:**

The primary outcome was Global Burden of Disease diagnosis category. Secondary outcomes were 28-day mortality rate, cause of death and healthcare delays, defined as delay in care-seeking, delay in reaching the healthcare facility and delay in appropriate treatment.

**Results:**

Communicable diseases were the most common presenting diagnoses among outpatients and among inpatients aged 1 month to 9 years. Non-communicable diseases and nutritional disorders were more common with increasing age. Few children presented with injuries. Newborn period (age <28 days) was associated with increased odds of death (OR 4.34 [95% CI 2.38 to 8.18], p<0.001, reference age 28 days–14 years) and there was no significant difference in odds of death between female vs male children (OR 1.12, 95% CI 0.6 to 2.04, p=0.72). 47 children died in the hospital (3.6%) and three (0.2%) died within 28 days of admission. Most children who died were <28 days old (n=32/50, 64%); leading diagnoses included neonatal sepsis/meningitis (n=13/50, 26%), neonatal encephalopathy (n=7/50, 14%) and lower respiratory tract infections (n=6/50, 12%). Delays in care-seeking (n=15) and in receiving appropriate treatment (n=12) were common.

**Conclusion:**

This study adds to sparse literature surrounding the epidemiology of disease and hospital outcomes for children with acute illness seeking healthcare in rural Pakistan and, in particular, among children aged 5–14 years. Further studies should include public and private hospitals within a single region to comprehensively describe patterns of care-seeking and interfacility transfer in district health systems.

**STRENGTHS AND LIMITATIONS OF THIS STUDY**

- This study aims to address limited empirical data describing disease burden and mortality rates among older children and adolescents seeking health services in low-income and middle-income countries.
- Community-based follow-up was used to characterise health outcomes for children following hospital discharge and contributing factors among children who died.
- This study was limited to the main district referral hospital in Sanghar district, Sindh; therefore, findings may not be generalisable to other areas in Pakistan, namely urban centres.
- Only age, sex, diagnosis and hospital disposition variables were available in the hospital registry data; therefore, it is not possible to characterise severity of illness, healthcare resource requirements, quality of care delivery and their impact on hospital outcomes.
- Further research is needed to understand population needs across the health system, including public and private hospitals and to describe patterns of care-seeking and interfacility transfer.

## Introduction

 It is estimated that between 0.7 and 1.1 million deaths occur annually worldwide among children and adolescents aged 5–14 years, largely due to preventable causes and with the overwhelming burden of mortality (98%) in low-income and middle-income countries (LMICs).^1^,^5^ While under-five deaths have significantly decreased significantly over the last two decades, older children have not experienced comparable reduction in mortality.^6^ Reliable epidemiological data are needed to ensure that gains in early childhood survival are met with strategies to promote health among older children and adolescents.

Recently, studies have described rates and causes of death among children and adolescents in LMICs,^1 3 7^ including in one using national vital statistics, large-scale surveys and population census data to produce global and national mortality estimates.^6^ These estimates were limited by scarce source data in most countries, with mortality data derived from the 0–5-year population or inferred from countries with adequate vital registration data or larger sample surveys.^6^ Another study analysed nationally representative mortality data among children 5–14 years in India, China, Brazil and Mexico,^3^ the only LMICs with 10 years of high-quality data. The leading causes of death were communicable diseases in India and injuries and non-communicable diseases in China, Brazil and Mexico.^3^ A 15-year review of trends in mortality in China among children 5 to 19 years used the national sample registration system to report nationally and subnationally representative rates and causes of death by sex and age group. Salient findings included higher all-cause mortality rates in rural regions, a greater decline in mortality rates among boys vs girls, resulting in a narrowing of observed sex differences in mortality, and an epidemiological transition in the leading cause of death among children 5–14 years from injuries to non-communicable diseases.^7^ These recent estimates were calculated using direct data sources; however, they may not be representative of other LMICs in Sub-Saharan Africa, Asia and the Americas.

In Pakistan, school-age children and young adolescents (5–14 years) make up 29% of the population and have an estimated mortality rate of 11.2 deaths per 1000 live births, 10-fold higher than in high-income countries.^8^ Structural determinants of health that may contribute to higher mortality rates among certain populations of school-age children and adolescents in Pakistan include poverty, unsafe sanitation, food insecurity and nutritional deficiencies, air pollution, regional conflict, frequency of natural disasters, illiteracy, poor road traffic and transport infrastructure, and gender inequality.^9^ The Pakistan National Emergency Department Surveillance (Pak-NEDS) study demonstrated that 85% of children presenting to urban, tertiary care general hospital emergency departments (ED) were aged 5 to 16 and disproportionately male (61 %).^10^ The most common presenting complaints were injuries, gastrointestinal complaints and respiratory complaints and ED mortality was 1%.^10^ This study did not examine the relationship between presenting complaint and mortality risk. In addition, although it is suspected that sociocultural factors may result in delayed healthcare seeking and excess mortality for girls in Pakistan, the study did not examine the relationship between sex or gender and mortality.^11 12^ The Pak-NEDS study, although not designed to be representative of the broader population in Pakistan, highlighted the preponderance of the 5–16 age group presenting to selected urban tertiary general EDs. Due to differences in healthcare accessibility and disease risk factors, the epidemiology of disease and outcomes for older children presenting to rural EDs may be quite different.

This study aims to compare the distribution of priority health conditions, 28-day mortality rates and causes of death across WHO age groups (0 to 14 years) and sex among children presenting with acute illness to Sanghar district headquarter (DHQ) hospital, Sindh province, Pakistan. We hypothesised that in rural Pakistan, injury and non-communicable diseases are the leading causes of presenting illness and death among children aged 10–14 years, while communicable diseases continue to represent the leading cause of illness and death among acutely ill children aged 5–9 years. Additionally, we hypothesised that mortality rates are equal or higher among girls compared with boys after adjusting for age.

## Methods

This is a prospective cohort study of consecutively recruited children presenting to the ED or outpatient department (OPD) at Sanghar DHQ hospital, Pakistan. Data was collected over a 9-month period between December 2019 and April 2020 and August 2020 and December 2020. Data collection was suspended between April and August 2020 due to the onset of the COVID-19 pandemic. Outcomes were ascertained at discharge from Sanghar DHQ and at 28-day community follow-up among children admitted to hospital.

### Setting

This study was carried out in Sanghar district of Sindh province, which has a population of 1.8 million, including 301 800 children under 5 years old, and is a largely rural, underdeveloped district.^13^ In Sindh province, under-five mortality is 56 per 1000 live births in urban areas and 93 per 1000 live births in rural areas compared with the national average of 74 per 1000 live births.^14^ Sanghar DHQ is a secondary care level, public sector institution. The hospital receives 1200 patient visits per day with 250 paediatric visits daily and has an average paediatric inpatient census of 26. There are 35 inpatient paediatric beds including a malnutrition ward, newborn nursery and general ward. There is no neonatal or paediatric intensive care unit and no formal trauma programme. The hospital mortality rate for children was unknown prior to initiation of this study.

### Participants

Based on feasibility considerations, we prospectively enrolled every third consecutive child aged 0–14 years presenting to the ED or OPD of the hospital and all children admitted to the inpatient department (IPD). Children were enrolled from the OPD as the majority of paediatric outpatients seek care for acute illness in the OPD on a first-come, first-served basis, while the ED is predominantly frequented by adults. Children presenting to the ED are routinely directed to the OPD for assessment and management. Children presenting for planned elective visits were excluded (eg, vaccinations, planned follow-up, routine well-child care and elective surgery).

### Data sources and variables

A structured electronic case record form was used to extract data from the outpatient (including ED and OPD) and inpatient department registries daily on a rolling basis throughout the enrolment period (online supplemental appendix 1). Demographic data (age, sex, place of residence), primary diagnosis, disposition from the ED or OPD and outcome at hospital discharge were recorded. Hospital discharge refers to ED or OPD discharge for patients who were not admitted. Age and sex were recorded as reported by caregivers. Gender was not recorded in the patient registries. At 28 days after hospital presentation, survival status of admitted children was determined by a community visit through the lady health worker (LHW) system. LHWs are government-funded, trained community healthcare providers who are each responsible for family planning and primary healthcare services for 150–200 homes.^15^ In the event of a death, a verbal and social autopsy (VASA) was conducted by trained study team members using the WHO 2016 verbal autopsy instrument and Institute for International Programmes of the Johns Hopkins University social autopsy questionnaire.^16 17^ The VASA consists of an interview with the child’s main caregiver regarding the child’s medical history and the details of the illness that led to death. Additional questions are posed to understand social and behavioural determinants of death including household, community and health system factors.^18^ Cause of death is determined from the VASA by independent classification of VASA interview findings by a trained study physician. Cause of death was determined in our study independently and in duplicate by two physicians following a training set; disagreements were resolved by consensus or by a third reviewer (Sajid Soofi) as needed.

The primary outcome was diagnosis category at hospital discharge classified according to the Global Burden of Disease 2017 groups: communicable diseases, non-communicable diseases, injuries, nutritional conditions, neonatal disorders, maternal conditions and ill-defined or cause unknown.^2^ The secondary outcomes were mortality at 28 days from hospital admission and cause of death. Of note, the initial study design included 28-day follow-up for all children in the OPD who were referred elsewhere for ongoing care and those who ‘left against medical advice’ to attempt to capture patients not admitted to hospital who may have been at higher risk of death. Due to feasibility challenges within the constraints of the COVID-19 pandemic and issues with the recording of home address in the OPD, follow-up was later amended to include only admitted children.

### Data management

All data was entered into a secure, electronic database developed in JAVA language with SQLite using handheld Android devices. Inpatient and outpatient records were linked using hospital record numbers. All data was de-identified for analysis.

### Data quality

Duplicate verification of the first 100 records was completed to identify entry errors and missing data. Implausible entries (eg, neonatal diagnoses in children >28 days of age or age >15 years) were verified against the registry and the individual patient chart as needed and set as missing if incomplete. Diagnoses entered as non-standard abbreviations or symptoms were set as missing if they could not be resolved. Following initial data entry for approximately 1000 outpatients, feedback was provided to the data collection teams to improve completeness and accuracy of data entry. At the conclusion of data collection, verification of inpatient study records was completed to determine the frequency of data entry errors.

### Sample size

For the primary analysis, 2962 patients were required to detect a 10% difference in diagnosis category distribution across four age categories and seven diagnosis categories at a significance level of 0.05 and power of 0.80. Sample size was calculated using formulae for χ square test of proportions described by Cohen and implemented in R using the Basic Functions for Power Analysis package.^19 20^

### Analysis

All analyses were performed using R statistical software (R Foundation for Statistical Computing, Vienna 2019). Recorded diagnoses were categorised according to subcategories of the Global Burden of Disease 2017 (communicable diseases, non-communicable diseases, injuries, nutritional conditions, neonatal disorders, maternal conditions and ill-defined or cause unknown).^3^ A trained reviewer analysed VASA transcripts using the Three Delays model.^21^ This framework characterises causes of mortality related to barriers in the health-seeking process into three possible sources of delay including (1) delay in care-seeking, (2) delay in reaching the healthcare facility and (3) delay in appropriate treatment.^21 22^ By understanding of the potential sources of delay, interventions can be targeted to improve health outcomes.

Results were summarised using descriptive statistics, disaggregated by sex (male and female) and WHO age category (<28 days, 28 days–11 months, 1–4 years, 5–9 years, 10–14 years) to allow comparison with existing disease burden estimates. Categorical variables were summarised as counts and proportions. The Fisher Exact Test was used to evaluate the association between age category and diagnosis category. Logistic regression was used to evaluate the association between age category and 28-day mortality after adjusting for sex. The distribution and frequency of missing data were summarised. It was assumed that the 28-day outcome may not be missing at random. Worst-case sensitivity analyses were planned if statistically significant differences in age, sex, diagnosis category or hospital disposition were identified among children with complete vs missing 28-day outcome. In worst case sensitivity analysis, we assumed that children whose discharge status was ‘left against medical advice’ and who were lost to follow-up had died at 28 days.

This study was approved by ethics review boards at Aga Khan University (2022-0979-21918), the Hospital for Sick Children (1000061181) and the University of Toronto (38710). Informed consent for participation was waived as data collection was limited to review of the patient registry and select medical records and all data collected was de-identified.

### Patient and public involvement

Patients and the public were not involved in the design, recruitment or conduct of this study.

## Results

Study enrollment occurred from 1 December 2019 to 17 March 2020 (period 1) and 1 August 2020 to 3 November 2020 (period 2). There was an interruption in data collection from 17 March 2020 to 1 August 2020 due to the onset of the COVID-19 pandemic and briefly due to a hospital strike in August 2020.

**Figure 1:**
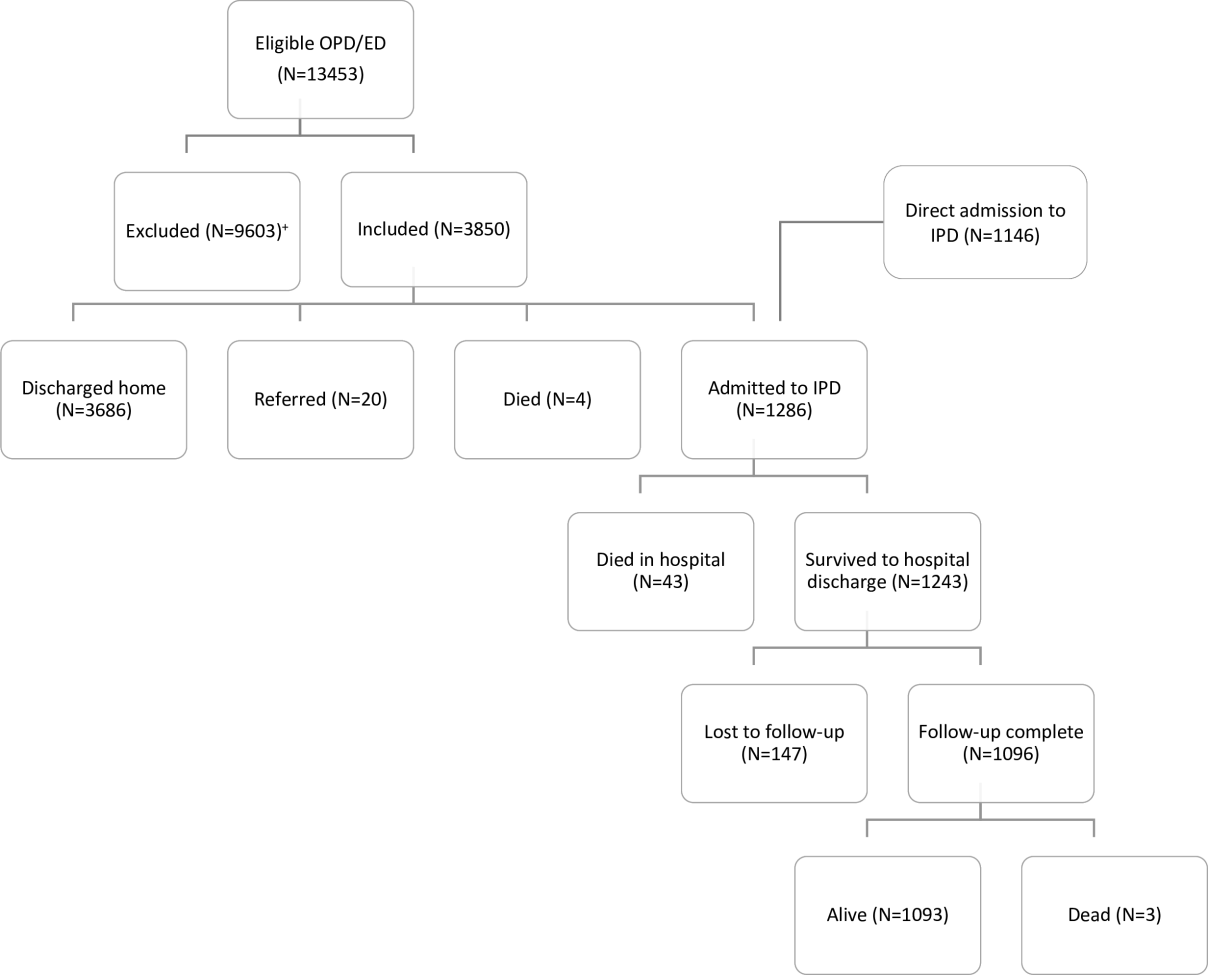
Flowchart of included patients. +: exclusions due to enrollment of every third consecutive patient and study interruption in the context of the COVID-19 pandemic.

Of 13 453 outpatients eligible over the study period, 3850 were included through enrollment of every third consecutive patient. All 1286 children admitted directly to the inpatient department or referred from the OPD were included; 1096 had complete follow-up at 28 days. 50 deaths were identified (4 in the OPD, 43 in the IPD and 3 at 28-day follow-up of IPD patients) and 19 verbal autopsies (38% of all deaths) were completed (figures1  2). Participant characteristics and missing data are summarised in tables1  2 and online supplemental appendices 1 and 2.

**Table 1:** Characteristics of children presenting to the emergency and outpatient department

	Overall(n=3850)	<1 month (n=158)	1–11 months (n=643)	1–4 years (n=1964)	5–14 years (n=1085)	P value‡
Diagnosis category, N (%)						<0.001*
Communicable diseases	3100 (80.5)	82 (51.9)	511 (79.5)	1628 (82.9)	879 (81)	
Injuries	49 (1.3)	1 (0.6)	7 (1.1)	20 (1.0)	21 (2)	
Neonatal disorders	32 (0.8)	29 (18.4)	3 (0.5)	0 (0)	0	
Non-communicable diseases	226 (5.9)	10 (6.3)	23 (3.6)	95 (4.8)	98 (9)	
Nutritional disorders	61 (1.6)	31 (19.6)	14 (2.2)	29 (1.5)	15 (1.4)	
Ill-defined	95 (2.5)	9 (5.7)	11 (1.7)	41 (2.1)	34 (3.1)	
Missing	287 (7.5)	24 (15.2)	74 (11.5)	151 (7.7)	38 (3.5)	
Leading diagnosis, N (%)^†^						
Upper respiratory tract infection	1541 (40)	35 (22.2)	185 (28.8)	805 (41)	516 (47.6)	
Lower respiratory tract infection	827 (21.5)	30 (19.0)	186 (28.9)	412 (21)	199 (18.3)	
Diarrheal diseases	612 (15.9)	15 (9.5)	128 (19.9)	333 (17)	136 (12.5)	
Hospital disposition, N (%)						<0.001*
Home	3686 (95.7)	117 (74.1)	581 (90.4)	1920 (97.8)	1068 (98.4)	
Admitted	140 (3.6)	32 (20.3)	55 (8.6)	40 (2)	13 (1.2)	
Referred	20 (0.5)	6 (3.8)	7 (1.1)	4 (0.2)	3 (0.3)	
Died	4 (0.1)	3 (1.9)	0 (0)	0 (0)	1 (0.1)	

* Fisher’s Exact Test, :top 3 leading diagnoses for each age and sex included, totals do not equal 100%, p values are reported for comparisons between age groups.

† Top 3 leading diagnoses for each age and sex included, totals do not equal 100%.

‡ p values are reported for comparisons between age groups.

**Table 2:** Characteristics of children admitted to the inpatient department

	Overall(n=1286)	<1 month (n=373)	1–11 months (n=345)	1–4 years (n=438)	5–9 years (n=114)	10–14 years (n=16)	P value‡
Diagnosis category							<0.001*
Communicable diseases	648 (50.4)	62 (16.6)	261 (75.6)	271 (61.9)	50 (43.9)	4 (25)	
Injuries	7 (0.5)	0 (0)	0	3 (0.7)	3 (2.6)	1 (6.3)	
Neonatal disorders	287 (22.4)	281 (75.3)	6 (1.7)	0 (0)	0 (0)	0 (0)	
Non-communicable diseases	97 (7.5)	6 (1.6)	11 (3.2)	44 (10)	32 (28.1)	4 (25)	
Nutritional disorders	116 (9)	3 (0.8)	33 (9.6)	63 (14.4)	12 (10.5	5 (31.3)	
Ill-defined	117 (9.1)	19 (5.1)	30 (8.7)	50 (11.4)	16 (14)	2 (12.5)	
Missing	14 (1.1)	2 (0.5)	4 (1.2)	7 (1.6)	1 (0.9)	0 (0)	
Leading diagnoses^†^							<0.001*
Anaemia	30 (2.3)	1 (0.3)	4 (1.2)	22 (5)	3 (2.6)	0 (0)	
Diarrheal diseases	322 (25)	22 (5.9)	128 (37.1)	152 (34.7)	19 (16.7)	1 (6.3)	
Epilepsy	2 (0.2)	0 (0)	0 (0)	1 (0.2)	0 (0)	1 (6.3)	
Febrile seizures	29 (2.3)	6 (1.6)	2 (0.6)	19 (4.3)	2 (1.8)	0 (0)	
Haemolytic disease and other neonatal jaundice	20 (1.6)	19 (5.1)	1 (0.3)	0 (0)	0 (0)	0 (0)	
Iron-deficiency anaemia	34 (2.6)	1 (0.3)	3 (0.9)	14 (3.2)	11 (9.6)	5 (31.3)	
Lower respiratory tract infection	243 (18.9)	35 (9.4)	103 (29.9)	69 (15.8)	23 (20.2)	3 (18.8)	
Neonatal encephalopathy due to birth asphyxia and trauma	84 (6.5)	80 (21.4)	3 (0.9)	1 (0.2)	0 (0)	0 (0)	
Neonatal preterm birth	22 (1.7)	22 (5.9)	0 (0)	0 (0)	0 (0)	0 (0)	
Neonatal sepsis and other neonatal infections	159 (12.4)	155 (41.6)	2 (0.6)	2 (0.5)	0 (0)	0 (0)	
Sepsis	32 (2.5)	0 (0)	17 (4.9)	13 (3)	1 (0.9)	1 (6.3)	
Severe acute malnutrition	82 (6.4)	2 (0.5)	30 (8.7)	49 (11.2)	1 (0.9)	0 (0)	
Snakebite	6 (0.5)	0 (0)	0 (0)	2 (0.5)	3 (2.6)	1 (6.3)	
Thalassaemias	50 (3.4)	0 (0)	5 (1.4)	14 (3.2)	28 (24.5)	3 (18.8)	
Upper respiratory tract infections	74 (5.8)	5 (1.3)	26 (7.5)	38 (8.7)	5 (4.4)	0 (0)	
Hospital disposition							<0.001*
Home	956 (74.3)	221 (59.2)	276 (80)	357 (81.5)	91 (79.8)	11 (68.8)	
Referred	229 (17.8)	107 (28.7)	48 (13.9)	49 (11.2)	21 (18.4)	5 (31.3)	
Died	43 (3.3)	28 (7.5)	6 (1.7)	9 (2.1)	0 (0)	0 (0)	
Left against medical advice	58 (4.5)	17 (4.6)	15 (4.3)	24 (5.5)	2 (1.8)	0 (0)	
Status at 28 days							0.21*
Alive	1093 (85)	307 (82.3)	299 (86.7)	379 (86.5)	94 (82.5)	14 (87.5)	
Dead	46 (3.6)	29 (7.8)	7 (2)	9 (2.1)	1 (0.9)	0 (0)	
Missing	147 (11.4)	37 (9.9)	39 (11.3)	50 (11.4)	19 (16.7)	2 (12.5)	

* Fisher’s Exact Test, :top 5 diagnoses for each age and sex included, totals do not equal 100%, p values are reported for comparisons between age group (aggregated sex).

† Top 5 diagnoses for each age and sex included, totals do not equal 100%.

‡ p values are reported for comparisons between age group (aggregated sex).

**Figure 2:**
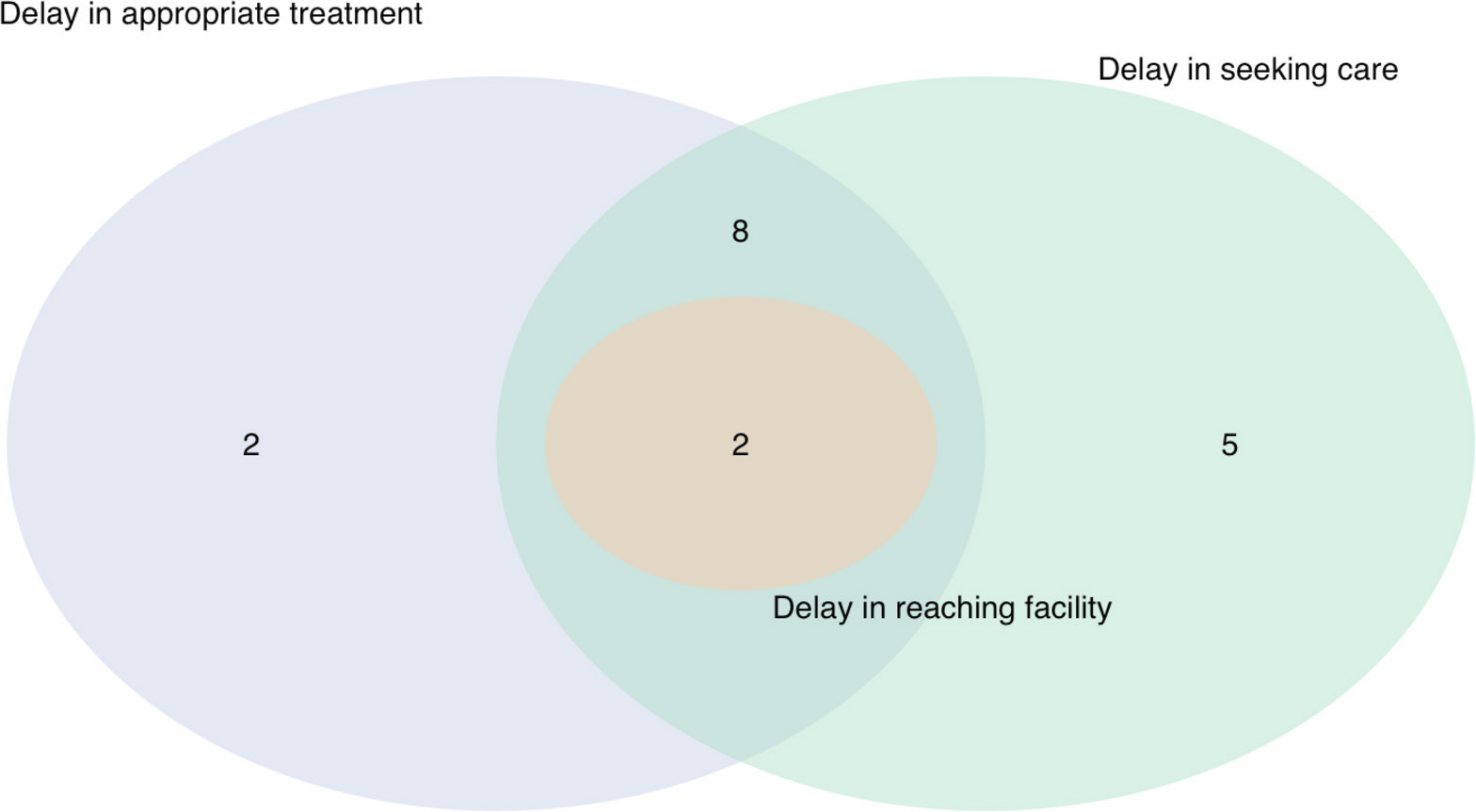
Sources of delay identified through verbal and social autopsy.

Children presenting to the outpatient and emergency department were predominantly aged 1–4 years (n=1964/3850, 69%) and 59% were male (n=2269/3850). Four children died in the OPD (n=4/3850, 0.1%). Communicable diseases were the most common diagnoses across all age groups in the outpatient department. Among children aged 28 days–14 years, there were statistically significant differences in diagnosis category by age category (p<0.001). Non-communicable diseases and nutritional disorders were more common among children 5–14 years compared with children 28 days–4 years.

The majority of children admitted to the IPD were aged less than 5 years (n=1156/1286, 90%) and 59% were male (n=764/1286). Among admitted children aged 28 days–14 years, there were statistically significant differences in diagnosis category according to age category (p<0.001). Communicable diseases were most common among children aged 1 month–9 years (n=582/897, 65%), while communicable diseases (n=4/16, 25%), nutritional disorders (n=5/16, 31%) and non-communicable diseases (n=4/16, 25%) were equally common among children 10–14 years. Non-communicable diseases were more common with increasing age (p<0.001). Few patients with injuries presented to the outpatient department (n=49/3779, 1.3%) or were admitted to the inpatient department (n=7/1286, 0.5%).

Of 1286 admitted children, 956 (74%) children were discharged alive, 229 (18%) were referred to another hospital, 58 (5%) left against medical advice, 43 (3%) died in hospital and of 1096 for whom 28-day follow-up was complete, 3 (0.2%) died at home after hospital discharge. Children who died were predominantly male (n=27, 58.7%), under 28 days old (n=32, 70%) and the most common admission diagnoses were neonatal sepsis and other neonatal infections (n=12, 26%) and neonatal encephalopathy due to birth asphyxia and trauma (n=7, 15%) (table 3).

**Table 3:** Characteristics of children who died by 28 days

	Total (n=50*)	<1 month (n=32)	1–11 months (n=7)	1–4 years (n=9)	5–14 years (n=2)
Female sex, N (%)	19 (38)	11 (34)	3 (43)	5 (56)	0 (0)
GBD diagnosis category, N (%)					
Communicable diseases	8 (16)	1 (3)	1 (14)	4 (44)	2 (100)
Neonatal disorders	25 (50)	25 (78)	0 (0)	0 (0)	0 (0)
Non-communicable diseases	3 (6)	1 (3)	1 (14.3)	1 (11)	0 (0)
Nutritional disorders	3 (6)	1 (3)	0 (0)	2 (22)	0 (0)
Ill-defined	10 (20)	3 (9)	5 (71.4)	222	0 (0)
Missing	1 (2)	1 (3)	0 (0)	0 (0)	0 (0)
Diagnosis, N (%)					
Anaemia	1 (2)	0 (0)	0 (0)	1 (11)	0 (0)
Congenital birth defects	1 (2)	1 (3)	0 (0)	0 (0)	0 (0)
Haemolytic disease and other neonatal jaundice	1 (2)	1 (3)	0 (0)	0 (0)	0 (0)
Hypothermia	1 (2)	1 (3)	0 (0)	0 (0)	0 (0)
Low birth weight	2 (4)	2 (6)	0 (0)	0 (0)	0 (0)
Lower respiratory tract infection	6 (12)	0 (0)	1 (14)	3 (33)	2 (100)
Meningitis	1 (2)	0 (0)	0 (0)	1 (11)	0 (0)
Neonatal encephalopathy due to birth asphyxia and trauma	7 (14)	7 (22)	0 (0)	0 (0)	0 (0)
Neonatal preterm birth	2 (4)	2 (6)	0 (0)	0 (0)	0 (0)
Neonatal sepsis and other neonatal infections	13 (26)	13 (41)	0 (0)	0 (0)	0 (0)
Sepsis	5 (10)	0 (0)	4 (57)	1 (11)	0 (0)
Severe acute malnutrition	3 (6)	1 (3)	0 (0)	2 (22)	0 (0)
Thalassaemia	1 (2)	0 (0)	1 (14)	0 (0)	0 (0)
Upper respiratory tract infections	1 (2)	1 (3)	0 (0)	0 (0)	0 (0)
Ill-defined†	4 (8)	2 (6)	1 (14)	1 (11)	0 (0)
Missing	1 (2)	1 (3)	0 (0)	0 (0)	0 (0)
Hospital length of stay, median (IQR)	0 (0-1)	0 (0-1)	0 (0-1)	0 (0)	0 (0)
Hospital disposition, N (%)					
Died	47 (94)	31 (97)	6 (86)	9 (100)	1 (100)
Home	2 (4)	1 (3)	1 (14)	0 (0)	0 (0)
Referred	1 (2)	0 (0)	0 (0)	0 (0)	1 (100)
Cause of death by verbal autopsy, N (%)	n=19	n=12	n=3	n=4	n=0
Acute respiratory infection	3 (6)	0 (0)	0 (0)	3 (33)	0 (0)
Meningitis	1 (2)	0 (0)	0 (0)	1 (11)	0 (0)
Neonatal diarrhoea	1 (2)	1 (3)	0 (0)	0 (0)	0 (0)
Neonatal pneumonia	1 (2)	1 (3)	0 (0)	0 (0)	0 (0)
Neonatal sepsis/meningitis	5 (26)	5 (16)	0 (0)	0 (0)	0 (0)
Not possible to determine	1 (2)	0 (0)	1 (14)	0 (0)	0 (0)
Perinatal asphyxia	3 (6)	3 (9)	0 (0)	0 (0)	0 (0)
Preterm birth complications	2 (4)	2 (6)	0 (0)	0 (0)	0 (0)
Sepsis	2 (4)	0 (0)	2 (29)	0 (0)	0 (0)
Missing	31 (62)	20 (62.5)	4 (57)	5 (56)	2 (100)
Sources of delay by VASA, N (%)‡	n=19	n=12	n=3	n=4	n=0
Delay in care-seeking	15 (30)	9 (28)	3 (43)	3 (33)	0 (0)
Delay in reaching the health facility	2 (4)	2 (6)	0 (0)	0 (0)	0 (0)
Delay in appropriate treatment	12 (24)	7 (22)	2 (29)	3 (33)	0 (0)
Missing	31 (62)	20 (63)	4 (57)	5 (56)	2 (100)

* 50 enrolled patients died: 47 in-hospital (43 IPD, 4 OPD) and 3 at 28-day follow-up.

† Ill-defined diagnoses: hypovolaemic shock, cardiogenic, normal baby.

‡ Multiple sources of delay possible for a given patient (Ccolumn totals do not sum to 100%).

GBDGlobal Burden of DiseaseVASAverbal and social autopsy

Age less than 28 days was associated with increased odds of death (OR 4.34 [95% CI 2.38 to 8.18], p<0.001, reference age 28 days to 14 years) and there was no significant difference in adjusted odds of death between male and female children (OR 1.12, 95% CI 0.6 to 2.04, p=0.72) (online supplemental appendix 6).

19 verbal autopsies were completed with the families of children who died (table 3). Seven caregivers refused verbal autopsy and 24 families could not be located to complete the VASA. Adjudicated causes of death identified through the VASA were neonatal sepsis/meningitis (n=6/19, 32%), perinatal asphyxia (n=3/19, 16%), acute respiratory infection (n=4/19, 21%), preterm birth complications (n=2/19, 11%), sepsis (n=2/19, 11%) and neonatal diarrhoea (n=1/19, 5%). Analysis using the Three Delays model revealed sources of delay for all but one child: delay in the decision to seek care in 15/19 cases (80%), delay in reaching the health facility in 2/19 cases (10%) and delay in receiving appropriate care at the facility in 12/19 cases (65%) (online supplemental material). 10 children experienced more than one source of delay. VASAs were not completed for the three children who died at home due to caregiver refusal; therefore no information regarding the circumstances of their deaths was available.

There was no missing data for age, sex or hospital outcome; 10% of diagnoses were missing or consisted of symptoms/syndromes. Duplicate verification of IPD records revealed few data entry errors which were corrected (diagnosis entry errors n=73/1286 [6%], age entry errors n=20/1286 [2%], disposition entry errors n=12/1286 [1%]). Among children who survived to hospital discharge, 12% had missing outcome data at 28 days (n=147/1243). There were no significant differences in age category (p=0.40), sex (p=0.20) or diagnosis category (p=0.90) among those with missing outcome at 28 days compared with complete cases (online supplemental appendix 5). A greater proportion of children with missing outcome were categorised as ‘left hospital against medical advice’ at discharge (p<0.001). Worst case sensitivity analysis, assuming that children who were discharged as ‘left against medical advice’ and lost to follow-up died, demonstrated results consistent with complete case analysis (online supplemental appendix 6); age <28 days was associated with greater odds of death (OR 2.58, 95% CI 1.58 to 4.21, p<0.001, reference age 28 days to 14 years) and there were no significant differences in adjusted odds of death between male and female children (OR 1.18, 95% CI 0.72, 1.93, p=0.50). There was no interaction between sex and age.

Between the two enrollment periods, December 2019 to March 2020 (period 1) and August 2020 to November 2020 (period 2), several differences were noted (online supplemental appendix 7). In the OPD, fewer patients presented per day in period 2 compared with period 1 (median 15 patients per day vs 30 per day) and a higher proportion of children had non-communicable diseases and nutritional disorders (p<0.001). A smaller proportion of children presented with respiratory tract infections in both the outpatient (43% in period 2 vs 67% in period 1, p<0.001) and inpatient department (3% in period 2 vs 31% in period 1, p<0.001). Few children presented to the OPD or were admitted with malaria throughout the study. There were no significant differences in age category or sex between the two enrollment periods in the outpatient department while in period 2, in the IPD, a greater proportion of admitted children were aged <1 month and 1–4 years (p<0.001). There were no significant differences in sex or diagnosis category over the enrollment periods among inpatients. After adjusting for age, there was a decreased odds of death among admitted children in period 2 (OR 0.43, 95% CI 0.23 to 0.79) compared with period 1.

## Discussion

Our study uses empirical data to address a knowledge gap regarding healthcare utilisation, diagnoses and outcomes in children, including school age children (5–9 years) and young adolescents (10–14 years) in Pakistan. The results of this study are in keeping with existing literature in Pakistan and elsewhere in South Asia in several ways. The burden of communicable diseases was high both in the inpatient and outpatient department among children 1–59 months and among older children 5–14 years in the outpatient department. In keeping with the global literature, neonates were at highest risk of death, primarily due to complications of prematurity, birth asphyxia and sepsis. Factors known to contribute to risk of neonatal death in Pakistan and globally include young maternal age, low maternal education level, socioeconomic disadvantage, short interpregnancy interval, inadequate antenatal care, place of delivery (eg, home vs facility birth), prematurity and small for gestational age birth weight.^23^,^25^ Our study findings are consistent with national surveillance data regarding cause of death for children under five in Pakistan and South Asia and two observational studies of presenting illness in an urban paediatric ED in Karachi.^914 26^,^30^ Similar to existing global estimates, the frequency of non-communicable diseases and nutritional disorders increased with age among children and adolescents in both outpatient and inpatient settings.^3 31^

There were a few notable differences in our data compared with limited published hospital data in Pakistan and Global Burden of Disease estimates for older children and adolescents. Children 5–14 years represented only 28% of outpatients and 1% of admitted children. This finding is consistent with the demographics of ED patients described in a single dedicated paediatric ED in Karachi, where children 5–13 years comprised 24% of enrolled patients, but contrasts with the larger Pak-NEDS study of 7 urban tertiary care general EDs in which children 5–16 years comprised 87% of those seeking care.^10^ The Pak-NEDS study authors hypothesised that in an urban setting, younger children are more likely to be brought to paediatric EDs and older children to general EDs.^10^ In Sanghar district, there is no paediatric hospital, which may explain the predominance of younger children in our study sample. In contrast to published estimates, injuries (unintentional injuries, transport injuries, interpersonal violence or conflict, or self-harm) were not a frequent cause of illness in the study cohort and no injury-related deaths were documented.^32 33^ South Asia has among the highest burden of unintentional injuries in children and adolescents globally with a higher-than-average incidence of injury-related death, particularly in conflict zones.^34^ In the Pak-NEDS study, injuries were the most common diagnoses (39%), predominantly among children 10–16 years.^10^ There may be several reasons why few children with injuries presented to Sanghar DHQ over the study period. First, Pakistan has a relatively underdeveloped and fragmented pre-hospital trauma care and transport system that relies on private-public partnership and is concentrated in urban areas.^35^ Children with severe injuries may succumb to their injuries prior to transport or may not be brought to hospital at all if it is deemed futile. Second, when private transport is secured, preference may be given by families to travel to tertiary care, private or dedicated paediatric facilities. Due to limited surveillance data outside of urban centres, little is known about the healthcare trajectory of children with injuries in rural Pakistan. Third, decreased incidence of road traffic accidents during the COVID-19 lockdown period may have contributed to fewer children presenting with injuries in the second enrollment period.^36 37^ Another notable difference in our study was that hospital and post-discharge mortality were relatively low compared with existing hospital data in Pakistan and other LMICs.^38^ In a paediatric ED in Karachi, reported ED mortality was 4%, 48-hour mortality was 12% and 14-day mortality was 20% among all children presenting for acute illness for whom follow-up was complete.^28^ It is now well-documented that post-discharge mortality in LMICs may be as high if not higher than hospital mortality due to failure to recognise severe illness, premature departure from hospital or secondary illness following the initial presentation.^38^ As we did not follow outcomes among patients who were discharged home from the OPD, it is not possible to determine from our study whether failure to recognise severe illness and inappropriate disposition may have contributed to underestimation of deaths. While we had initially planned to conduct 28-day follow-up for all children in the OPD who were referred elsewhere (n=20) or recorded as ‘left against medical advice’ (n=0), this was revised due to feasibility concerns and incomplete documentation of home address in the OPD. Lack of post-discharge follow-up among children attending the OPD who were referred or discharged home may have contributed to underestimation of mortality, particularly if children at high risk of death or imminently dying were referred elsewhere. Among the admitted children who were lost to follow-up at 28 days, it is striking that there was a greater proportion whose hospital disposition was recorded as ‘left against medical advice’. If these children left hospital due to perceived futility in the setting of severe illness or due to incentive to pursue care in another institution, this may have contributed to underestimation of post-discharge mortality. In addition, further study is needed to determine whether children with more severe illness in Sanghar district are travelling to reach dedicated paediatric facilities, preferentially attending private facilities, or simply not brought to medical attention in time and succumbing to their illness at home.

We did not observe an association between sex and mortality; however, male children were disproportionately represented in the study sample relative to the population male:female sex ratio of 1.06:1. Among male neonates, this may be explained by known biological susceptibility to infection and post-birth complications which increase neonatal male healthcare seeking and mortality risk.^39 40^ Gender-based analysis in our study is limited by the absence of gender identification in the patient registry. Caregiver-reported sex likely corresponds to perceived gender and associated potential for gender bias. Pakistan continues to have excess mortality among girls under five and 5–9 years of age.^12 41^ Beyond the neonatal period, the disproportionate representation of male children in our study and similar mortality rate between male and female children raises concerns about the impact of gender bias on healthcare seeking for girls.^42^

Through analysis of verbal and social autopsies, sources of delay were characterised among children who died. Delay in the decision to seek care was most common, followed by delay in receiving appropriate care at the facility, whereas delay in reaching the facility was identified in only two cases. 10 children experienced more than one source of delay, consistent with existing literature describing the inter-connectedness of the Three Delays across the care continuum.^17 43^ While caregivers may recognise signs of severe illness, the decision to seek care is often delayed for many reasons including preference for home or traditional remedies, cost, lack of transportation, distance to the facility and lack of caregiver autonomy in decision-making.^43 44^ Delay in care-seeking contributes to increased severity of illness at the time of presentation to hospital, at which time the condition may no longer be reversible and this is compounded by treatment delay or delivery of poor quality care. Negative user experiences and perceived low quality of care at a given facility may contribute to future delays in the decision to seek formal care.^45^ Moreover, caregivers may elect to bypass a facility altogether in favour of private health services or tertiary hospital care, potentially contributing to increase in transport time to reach definitive care.^45^

Significant differences noted prior to and following the onset of the COVID-19 pandemic included an overall decrease in the number of patients presenting to hospital and a decrease in the proportion of children presenting with communicable diseases from August to November 2020, particularly respiratory infections. This is consistent with changes in communicable disease patterns documented in Pakistan from February 2020 onward relative to prior years.^46^ National surveillance data demonstrated a decrease in incidence of reported non-COVID-19 upper respiratory tract infections, pneumonia and enteric infections from March 2020 to February 2021 compared with similar periods in the preceding 2 years.^46^ Missaghi *et al* describe an inverse relationship between increased incidence of COVID-19 infection and the incidence of other communicable diseases with each wave of infection. While changes in the epidemiology of non-COVID-19 communicable diseases explain some of the findings of our study, there remains concern that decreased care-seeking may also have contributed to the reduced number of patients brought to hospital and a difference in typical disease epidemiology. For example, in the rainy and retreating monsoon season from August to October, a typical seasonal increase in incidence of malaria occurred in Pakistan; however, this pattern was not observed in our study cohort.^46^ Lastly, due to the COVID-19-related interruptions in data collection between April and August 2020, we are unable to describe disease epidemiology during the peak hot season, typically associated with increased incidence of enteric infections and heat-related illness.^46 47^

Our study has several strengths. Data collection was embedded in routine care practices and community follow-up to avoid adding burden to existing high clinical workload in a resource-limited setting. Data quality was ensured through duplicate verification of records and additional training of data abstractors as needed. The frequency of missing data was low, and follow-up was nearly complete. Finally, our study included older children and young adolescents who have been historically under-represented in the global health literature.

Limitations of the study include the potential for erroneous data entries, under-reporting of patient deaths. This was mitigated through duplicate data verification of the IPD dataset; however, it remains possible that errors were missed or deaths reported as ‘left against medical advice’. We would expect these misclassification errors to occur at random across ages and diagnosis categories. Follow-up data was assumed to be missing at random and there were no statistically significant differences in age, sex or diagnosis category between those with complete vs incomplete follow-up. Nevertheless, it is possible that families of children who died would be more likely to be lost to follow-up leading to underestimation of mortality across all age groups. In addition, the study was limited to the main district referral hospital and only age, sex, diagnosis and hospital disposition variables were available in the hospital registry data. From this data, it is not possible to characterise severity of illness, healthcare resource requirements, quality of care delivery and their impact on hospital outcomes. There is also potential for incomplete caregiver recall of events during VASA interviews which may contribute to under-reporting of sources of delay contributing to a child’s death. This limitation was mitigated by trained interviewers using a structured, validated process with an emphasis on eliciting a narrative, chronological summary of events. Given discrepancies between our study findings and limited published data for urban hospital settings in Pakistan, we cannot generalise our findings to other regions in Pakistan. Finally, while our study findings suggest some early effects of the COVID-19 pandemic on infectious disease epidemiology and care-seeking, the enrollment period was too short to capture longer-term effects including the impact of the pandemic response on routine immunisation uptake and emergence of vaccine-preventable disease.

## Conclusion

This study adds to scarce literature surrounding the epidemiology of disease and hospital outcomes for children with acute illness seeking healthcare in Pakistan and, in particular, children aged 5–14 years. This study found differences in presenting diagnosis based on age among children with acute illness seeking healthcare in Pakistan. Delays in care-seeking and delivery of appropriate treatment occurred in most patients who died. To curb preventable mortality in settings such as the Sanghar DHQ, targeted neonatal and paediatric emergency and critical care interventions are needed.^48^,^52^ Improved understanding of case mix and causes of death as described in this study can inform future service delivery planning within the setting of limited resources. Further studies should include public and private hospitals within a single region to comprehensively describe patterns of care-seeking and interfacility transfer. Specific attention is needed to further characterise the burden of non-communicable diseases, nutritional disorders and injuries among older children and their particular healthcare needs.

## supplementary material

10.1136/bmjopen-2023-082255 online supplemental file 1

## Data Availability

Data are available upon reasonable request.
