# Supplementary material for: The spectrum of acute illness and mortality of children and adolescents presenting to emergency services in Sanghar district hospital, Pakistan: a prospective cohort study
Source: BMJ Open. 2024 Aug 22;14(8):e082255. doi: 10.1136/bmjopen-2023-082255 (PMC11344522; doi:10.1136/bmjopen-2023-082255)
Supplement: online supplemental file 1 [file bmjopen-14-8-s001.pdf]

Appendix 1 Data variables collected

Variable	Response Frame
Study identifier	Numeric value
Date of admission	DD-MM-YYYY
Date of discharge	DD-MM-YYYY
Age category	< 1 month; 1–11 months, 1–4 years, 5–9 years, 10–14 years
Sex	Female, male
Most responsible diagnosis	Drop-down selection of Global Burden of Disease diagnoses
Other diagnosis	Free text
Disposition	Home, admitted, referred, left against medical advice, died
Status at 28 days	Alive, died, missing

Appendix 2 Characteristics of children presenting to the emergency and outpatient department

	Overall		< 1 month		1–11 months		1–4 years		5–14 years		
	**Male (N=2269)**	**Female (N=1581)**	**Male (N=104)**	**Female (N=54)**	**Male (N=382)**	**Female (N=261)**	**Male (N=1149)**	**Female (N=815)**	**Male (N=634)**	**Female (N=451)**	**p value**
**Diagnosis category, N(%)**											<0.001^
Communicable diseases	1804 (79.5)	1296 (82)	55 (52.9)	27 (50)	307 (80.4)	204 (78.2)	940 (81.8)	688 (84.4)	502 (79.2)	377 (83.6)	
Injuries	40 (1.8)	9 (0.6)	1 (1)	0 (0)	4 (1)	3 (1.1)	17 (1.5)	3 (0.4)	18 (2.8)	3 (0.7)	
Neonatal disorders	26 (1.1)	6 (0.4)	23 (22.1)	6 (11.1)	3 (0.8)	0 (0)	0 (0)	0 (0)	0 (0)	0 (0)	
Non communicable diseases	141 (6.2)	85 (5.4)	4 (3.8)	6 (11.1)	13 (3.4)	10 (3.8)	60 (5.2)	35 (4.3)	64 (10.1)	34 (7.5)	
Nutritional disorders	32 (1.4)	29 (1.8)	2 (1.9)	1 (1.9)	7 (1.8)	7 (2.7)	15 (1.3)	14 (1.7)	8 (1.3)	7 (1.6)	
Ill-defined	57 (2.5)	38 (2.4)	6 (5.8)	3 (5.6)	6 (1.6)	5 (1.9)	20 (1.7)	21 (2.6)	25 (3.9)	9 (2)	
Missing	169 (7.4)	118 (7.5)	13 (12.5)	11 (20.4)	42 (11)	32 (12.3)	97 (8.4)	54 (6.6)	17 (2.7)	21 (4.7)	
**Leading diagnosis, N(%)**+											
Upper respiratory tract infection	888 (39.1)	653 (41.3)	23 (22.1)	12 (22.2)	115 (30.1)	70 (26.8)	475 (41.3)	330 (40.5)	275 (43.4)	241 (53.4)	
Lower respiratory tract infection	495 (21.8)	332 (21)	19 (18.3)	11 (20.4)	110 (28.8)	76 (29.1)	243 (21.1)	169 (20.7)	123 (19.4)	76 (16.9)	
Diarrheal diseases	349 (15.4)	263 (16.6)	12 (11.5)	3 (5.6)	73 (19.1)	55 (21.1)	175 (15.2)	158 (19.4)	89 (14.0)	47 (10.4)	
**Hospital Disposition, N (%)**											<0.001^
Home	2165 (95.4)	1521 (96.2)	81 (77.9)	36 (66.7)	341 (89.3)	240 (92)	1121 (97.6)	799 (98)	622 (98.1)	446 (98.9)	
Admitted	86 (3.8)	54 (3.4)	15 (14.4)	17 (31.5)	37 (9.7)	18 (6.9)	26 (2.3)	14 (1.7)	8 (1.3)	5 (1.1)	
Referred	14 (0.6)	6 (0.4)	5 (4.8)	1 (1.9)	4 (1)	3 (1.1)	2 (0.2)	2 (0.2)	3 (0.5)	0 (0)	
Died	4 (0.2)	0 (0)	3 (2.9)	0 (0)	0 (0)	0 (0)	0 (0)	0 (0)	1 (0.2)	0 (0)	

^ Fisher's Exact Test, +:top 3 leading diagnoses for each age and sex included, totals do not equal 100%, #p values are reported for comparisons between age groups (aggregated sex)

Appendix 3 Characteristics of children admitted to the inpatient department

	Overall		< 1 month		1–11 months		1–4 years		5–9 years		10 - 14 years		
	Male (N=764)	Female (N=522)	Male (N=234)	Female (N=139)	Male (N=214)	Female (N=131)	Male (N=245)	Female (N=193)	Male (N=62)	Female (N=52)	Male (N=9)	Female (N=7)	p value#
**Diagnosis Category**													<0.001^
Communicable diseases	371 (48.6)	277 (53.1)	37 (15.8)	25 (18)	161 (75.2)	100 (76.3)	143 (58.4)	128 (66.3)	27 (43.5)	23 (44.2)	3 (33.3)	1 (14.3)	
Injuries	4 (0.5)	3 (0.6)	0 (0)	0 (0)	0 (0)	0 (0)	2 (0.8)	1 (0.5)	1 (1.6)	2 (3.8)	1 (11.1)	0 (0)	
Neonatal disorders	186 (24.3)	101 (19.3)	181 (77.4)	100 (71.9)	5 (2.3)	1 (0.8)	0 (0)	0 (0)	0 (0)	0 (0)	0 (0)	0 (0)	
Non communicable diseases	66 (8.6)	31 (5.9)	4 (1.7)	2 (1.4)	9 (4.2)	2 (1.5)	31 (12.7)	13 (6.7)	20 (32.3)	12 (23.1)	2 (22.2)	2 (28.6)	
Nutritional disorders	63 (8.2)	53 (10.2)	1 (0.4)	2 (1.4)	17 (7.9)	16 (12.2)	39 (15.9)	24 (12.4)	5 (8.1)	7 (13.5)	1 (11.1)	4 (57.1)	
Ill-defined	69 (9)	48 (9.2)	11 (4.7)	8 (5.8)	21 (9.8)	9 (6.9)	26 (10.6)	24 (12.4)	9 (14.5)	7 (13.5)	2 (22.2)	0 (0)	
Missing	5 (0.7)	9 (1.7)	0 (0)	2 (1.4)	1 (0.5)	3 (2.3)	4 (1.6)	3 (1.6)	0 (0)	1 (1.9)	0 (0)	0 (0)	
**Leading diagnoses+**													<0.001^
Anemia	19 (2.5)	11 (2.1)	1 (0.4)	0 (0)	2 (0.9)	2 (1.5)	13 (5.3)	9 (4.7)	3 (4.8)	0 (0)	0 (0)	0 (0)	
Diarrheal diseases	187 (24.5)	135 (25.9)	14 (6.0)	8 (5.8)	73 (34.1)	55 (42.0)	86 (35.1)	66 (34.2)	13 (21)	6 (11.5)	1 (11.1)	0 (0)	
Epilepsy	2 (0.3)	0 (0)	0 (0)	0 (0)	0 (0)	0 (0)	1 (0.4)	0 (0)	0 (0)	0 (0)	1 (11.1)	0 (0)	
Febrile seizures	18 (2.4)	11 (2.1)	4 (1.7)	2 (1.4)	0 (0)	2 (1.5)	13 (5.3)	6 (3.1)	1 (1.6)	1 (1.9)	0 (0)	0 (0)	
Hemolytic disease and other neonatal jaundice	16 (2.1)	4 (0.8)	15 (6.4)	4 (2.9)	1 (0.5)	0 (0)	0 (0)	0 (0)	0 (0)	0 (0)	0 (0)	0 (0)	
Iron-Deficiency Anemia	18 (2.4)	16 (3.1)	1 (0.4)	0 (0)	2 (0.9)	1 (0.8)	9 (3.7)	5 (2.6)	5 (8.1)	6 (11.5)	1 (11.1)	4 (57.1)	
Lower respiratory tract infection	131 (17.1)	102 (19.5)	20 (8.5)	15 (10.8)	69 (32.2)	34 (26.0)	31 (12.7)	38 (19.7)	9 (14.5)	14 (26.9)	2 (22.2)	1 (14.3)	
Neonatal encephalopathy due to birth asphyxia and trauma	58 (7.6)	26 (5)	55 (23.5)	25 (18)	2 (0.9)	1 (0.8)	1 (0.4)	0 (0)	0 (0)	0 (0)	0 (0)	0 (0)	
Neonatal Preterm Birth	16 (2.1)	6 (1.1)	16 (6.8)	6 (4.3)	0 (0)	0 (0)	0 (0)	0 (0)	0 (0)	0 (0)	0 (0)	0 (0)	
Neonatal sepsis and other neonatal infections	94 (12.3)	65 (12.5)	91 (38.9)	64 (46.0)	2 (0.9)	0 (0)	1 (0.4)	1 (0.5)	0 (0)	0 (0)	0 (0)	0 (0)	
Sepsis	20 (2.6)	12 (2.3)	0 (0)	0 (0)	13 (6.1)	4 (3.1)	5 (2)	8 (4.1)	1 (1.6)	0 (0)	1 (11.1)	0 (0)	
Severe acute malnutrition	45 (5.9)	37 (7.1)	0 (0)	2 (1.4)	15 (7.0)	15 (11.5)	30 (12.2)	19 (9.8)	0 (0)	1 (1.9)	0 (0)	0 (0)	
Snakebite	3 (0.4)	3 (0.6)	0 (0)	0 (0)	0 (0)	0 (0)	1 (0.4)	1 (0.5)	1 (1.6)	2 (3.8)	1 (11.1)	0 (0)	
Thalassemias	33 (4.3)	17 (3.3)	0 (0)	0 (0)	5 (2.3)	0 (0)	11 (4.5)	3 (1.6)	16 (25.8)	12 (23.1)	1 (11.1)	2 (28.6)	
Upper respiratory tract infections	42 (5.5)	32 (6.1)	3 (1.3)	2 (1.4)	17 (7.9)	9 (6.9)	20 (8.2)	18 (9.3)	2 (3.2)	3 (5.8)	0 (0)	0 (0)	
**Hospital Disposition**													<0.001^
Home	561 (73.4)	395 (75.7)	133 (56.8)	88 (63.3)	167 (78)	109 (83.2)	210 (85.7)	147 (76.2)	45 (72.6)	46 (88.5)	6 (66.7)	5 (71.4)	
Referred	151 (19.8)	78 (14.9)	75 (32.1)	32 (23)	36 (16.8)	12 (9.2)	22 (9)	26 (13.5)	15 (24.2)	6 (11.5)	3 (33.3)	2 (28.6)	
Died	24 (3.1)	19 (3.6)	17 (7.3)	11 (7.9)	3 (1.4)	3 (2.3)	4 (1.6)	5 (2.6)	0 (0)	0 (0)	0 (0)	0 (0)	
Left against medical advice	28 (3.7)	30 (5.7)	9 (3.8)	8 (5.8)	8 (3.7)	7 (5.3)	9 (3.7)	15 (7.8)	2 (3.2)	0 (0)	0 (0)	0 (0)	
**Status at 28-days**													0.21^
Alive	657 (86)	436 (83.5)	192 (82.1)	115 (82.7)	190 (88.8)	109 (83.2)	215 (87.8)	164 (85)	52 (83.9)	42 (80.8)	8 (88.9)	6 (85.7)	
Dead	27 (3.5)	19 (3.6)	18 (7.7)	11 (7.9)	4 (1.9)	3 (2.3)	4 (1.6)	5 (2.6)	1 (1.6)	0 (0)	0 (0)	0 (0)	
Missing	80 (10.5)	67 (12.8)	24 (10.3)	13 (9.4)	20 (9.3)	19 (14.5)	26 (10.6)	24 (12.4)	9 (14.5)	10 (19.2)	1 (11.1)	1 (14.3)	

^ Fisher's Exact Test, +:top 5 diagnoses for each age and sex included, totals do not equal 100%, #p values are reported for comparisons between age group (aggregated sex)

Appendix 4 Association between age category, sex, and 28-day mortality among children admitted to the IPD

Predictors	Odds Ratios	Standard Error	95% Confidence interval	p value
(Intercept)	0.02	0.006	0.01 – 0.03	<0.001
Age < 28 days (Ref 28 days – 14 years)	4.34	1.36	2.38 – 8.18	<0.001
Female sex (Ref: Male)	1.12	0.35	0.60 – 2.04	0.72
Observations	1131 (46 events)			
AIC	368			
AUROC	0.68			
Hosmer Lemeshow p value	0.82			

Appendix 5 Characteristics of children lost to follow-up at 28 days

	Total (N=1243)	28-day follow-up completed (N=1096)	Lost to follow-up (N=147)	p value
**Age Category, N(%)**				0.39^
< 1 month	345 (27.8)	308 (28.1)	37 (25.2)	
1 - 11 months	339 (27.3)	300 (27.4)	39 (26.5)	
1 - 4 years	429 (34.5)	379 (34.6)	50 (34.0)	
5 - 9 years	114 (9.2)	95 (8.7)	19 (12.9)	
10 - 14 years	16 (1.3)	14 (1.3)	2 (1.4)	
**Sex**				0.22*
Male	740 (59.5)	660 (60.2)	80 (54.4)	
Female	503 (40.5)	436 (39.8)	67 (45.6)	
**GBD Diagnosis Category, N(%)**				0.85^
Communicable diseases	642 (51.6)	575 (52.5)	67 (45.6)	
Injuries	7 (0.6)	7 (0.6)	0 (0)	
Neonatal disorders	263 (21.2)	230 (21.)	33 (22.4)	
Non communicable diseases	96 (7.7)	86 (7.8)	10 (6.8)	
Nutritional disorders	113 (9.)	97 (8.9)	16 (10.9)	
Ill-defined	108 (8.7)	89 (8.1)	19 (12.9)	
Missing	14 (1.1)	12 (1.1)	2 (1.4)	
**Disposition at Hospital Discharge, N (%)**				<0.001
Home	956 (76.9)	863 (78.7)	93 (63.3)	
Referred	229 (18.4)	199 (18.2)	30 (20.4)	
Left against medical advice	58 (4.7)	34 (3.1)	24 (16.3)	

^ Fisher's Exact Test, * Chi Square Test

Appendix 6 Worst case and complete case analysis of the association between age, sex, and 28-day mortality

Analysis	Intercept OR (95% CI, p value)	Age <28 days [ref. Age 28 days-14 years] OR (95% CI, p value)	Female sex [ref. Male sex] OR (95% CI, p value)
**Complete case analysis**	0.02 (0.01-0.03, p<0.001)	4.39 (2.40-8.26, p<0.001)	1.12 (0.6-2.04, p<0.7)
**Worst case analysis**	0.04 (0.03-0.06, p<0.001)	2.58 (1.58-4.21, p<0.001)	1.18 (0.72-1.93, p<0.5)

Appendix 7 Patient characteristics in the inpatient department prior to and following onset of the COVID-19 pandemic

	Total (N=1286)	December 2019 - March 2020 (N=666)	August 2020 - November 2020 (N=620)	p value
**Age Category, N (%)**				<0.001^
< 1 month	373 (29)	182 (27.3)	191 (30.8)	
1 - 11 months	345 (26.8)	202 (30.3)	143 (23.1)	
1 - 4 years	438 (34.1)	184 (27.6)	254 (41)	
5 - 9 years	114 (8.9)	83 (12.5)	31 (5)	
10 - 14 years	16 (1.2)	15 (2.3)	1 (0.2)	
**Sex, N (%)**				0.5*
Male	764 (59.4)	390 (58.6)	374 (60.3)	
Female	522 (40.6)	276 (41.4)	246 (39.7)	
**GBD Diagnosis Category, N (%)**				0.3*
Communicable diseases	648 (50.4)	344 (51.7)	304 (49)	
Injuries	7 (0.5)	5 (0.8)	2 (0.3)	
Neonatal disorders	287 (22.3)	139 (20.9)	148 (23.9)	
Non communicable diseases	97 (7.5)	53 (8)	44 (7.1)	
Nutritional disorders	116 (9)	56 (8.4)	60 (9.7)	
Ill-defined	117 (9.1)	61 (9.2)	56 (9)	
Missing	14 (1.1)	8 (1.2)	6 (1)	
**Disposition, N (%)**				<0.001*
Home	956 (74.3)	446 (67)	510 (82.3)	
Referred	229 (17.8)	149 (22.4)	80 (12.9)	
Died	43 (3.3)	26 (3.9)	17 (2.7)	
Left against medical advice	58 (4.5)	45 (6.8)	13 (2.1)	

^ Fisher's Exact Test, * Chi Square Test
