# Supplementary material for: The spectrum of acute illness and mortality of children and adolescents presenting to emergency services in Sanghar district hospital, Pakistan: a prospective cohort study
Source: BMJ Open. 2024 Aug 22;14(8):e082255. doi: 10.1136/bmjopen-2023-082255 (PMC11344522; doi:10.1136/bmjopen-2023-082255)
Supplement: Supplementary file 2 [file bmjopen-2023-082255.reviewer_comments.pdf]

PEER REVIEW HISTORY

BMJ Open publishes all reviews undertaken for accepted manuscripts. Reviewers are asked to complete a checklist review form (**http://bmjopen.bmj.com/site/about/resources/checklist.pdf**) and are provided with free text boxes to elaborate on their assessment. These free text comments are reproduced below.

ARTICLE DETAILS

**TITLE (PROVISIONAL)**	The Spectrum of acute illness and mortality of children and adolescents presenting to emergency services in Sanghar district hospital, Pakistan: A prospective cohort study
**AUTHORS**	Muttalib, Fiona; Memon, Zahid Ali; Muhammad, Shah; Soomro, Asif; Khan, Samia; Bano, Shazia; Jawwad, Muhammad; Soofi, Sajid; Hansen, B; Adhikari, Neill; Bhutta, Zulfiqar

VERSION 1 – REVIEW

**REVIEWER**	Ante-Ardila,
**REVIEW RETURNED**	15-Dec-2023

**GENERAL COMMENTS**	I want to congratulate the authors for the effort and work done. It is a very important issue, given that, as mentioned, there is not enough information on infant mortality in low-middle-income countries, and there is not enough clarity on the association between acute diseases and mortality.<br><br>I consider you have done a good job, however I have some recommendations and corrections that I suggest:<br>*Abstract:<br>- Add a little more background in the introduction to know the context and importance of this article<br><br>*Strengths and limitations: It is not clear what the limitations are, I suggest mentioning the possible biases that could have occurred, and what they did to avoid them<br><br>*I suggest checking the grammar in English<br><br>*Methods:<br>- The inclusion and exclusion criteria are not clear: were only those who were going to planned medical appointments excluded?<br>The inclusion criteria were all children between 0-14 who consulted, including neonates, and patients with comorbidities?<br>"A structured case record form was used to extract data from the outpatient (including ED and OPD)..." I suggest adding the case record form as a supplementary file<br>- "Improbable entries (for example, neonatal diagnoses in children >28 days of age or >15 years) were compared to the individual patient record and medical record as necessary and were marked as missing if incomplete." When participants were mentioned you described patients between 0 and 14 years old, you did not mention that neonates <28 days old were excluded. It is not clear if they were included or not.<br>- I suggest adding the sample size calculation as a supplementary file

	analysis:<br>- I suggest that the methodology and analysis be reviewed by a statistician, since it is not my field of expertise.<br>- You mention that the study was approved by the ethics review board. But you do not mention whether any type of consent and assent was requested from the parents, guardians and/or patients participating in the study, which is important as it is a prospective cohort study.<br><br>*Results and discussion::<br>- I suggest explaining what diagnoses were considered communicable diseases and non-communicable diseases.<br>- Of the patients who died at home, did they show clinical recovery upon discharge and subsequently became complicated? or since discharge they showed signs of complications?<br>- I consider reinforcing the discussion, for example, why do you think neonates were the most affected group, what are the risk factors for that age group?<br>- I suggest making more comparisons with respect to the literature, not only from low-income countries, but also from developed countries.<br><br>I congratulate you for the effort made, it is a very important work, I consider it requires some corrections for its consideration to be published. I would like to see the corrections and the publication of the article in the future. Thank you for the valuable effort of contributing to the medical literature.

**REVIEWER**	Ibtihal Abdelgadir<br>Sidra Medical and Research Center, Doha , pediatrics
**REVIEW RETURNED**	24-Dec-2023

**GENERAL COMMENTS**	Dear authors,<br><br>I would congratulate the authors for this good study,<br><br>Few points to be addressed and considered for improvements.<br><br>Abstract:<br>“this study found differences in presenting diagnosis based on age among children with acute illness seeking healthcare in Pakistan.” This study is done in one hospital/center, with selective inclusions, so would not be reasonable to have that overall conclusion to include all healthcare in Pakistan.<br>Methodology<br>“we enrolled every third consecutive child aged 0–14 years presenting to the ED or OPD of the hospital and all children admitted to the inpatient department” I just wondered why not included all patients attended the ED or OPD, as that will get more robust results, especially when the results was stratified into few parameters including age and sex.<br><br><br>VASA is done by trained personnel, please can you explain briefly the VASA and the “Three delay model,”<br><br>“then that was followed by case of death assignment “ explanation for the need of this step of assessment, as currently look like an extra step, that is not needed.

	Data collection was done by accessing the patients' records, mainly retrospectively, can we clarify the prospective element of this study please<br><br>Results<br>Data within the table might be better with some modifications as not easy to understand.<br><br>Please consider these comments for guidance..<br><br>Table 1<br><br>4 patients died in OPD, this is quite unusual as critically unwell patients, would be usually assessed in an emergency department, or to be shifted to a higher facility, unless death on arrival, please explain more.<br><br>Table 2<br>The calculation of the number of patients who died, not very clear, two calculations were presented, for deposition and at 28 days. What is the cut off for both, and why not to add them together as overall mortality?/<br><br>P values presented for all tables, assessed which variables, as there are multiple variables presented within the table, among different age groups and both sexes, please clarify?/<br><br>The conclusion about the mortality in children less than 28 days, associated trauma diagnosis can be discussed, however, the hospital under assessment on this study is not mentioned whether there is a neonatal unit/intensive care and/or the level of trauma service delivered. That would be part of the limitation of the study.

**REVIEWER**	Adrian Esterman<br>University of South Australia, School of Nursing and Midwifery
**REVIEW RETURNED**	14-Mar-2024

**GENERAL COMMENTS**	Under the sample size section, please provide the effect size used.<br>In the tables, please right adjust columns, and standardise the number of decimal points used.<br>Given the study design, why have you not used log binomial models rather than logistic regression models and obtained rate ratios rather than odds ratios?

VERSION 1 – AUTHOR RESPONSE

Reviewer: 1
Dr. Natalia Ante-Ardila, Hospital Universitario de la Fundación Santa Fe de Bogotá
Comments to the Author:

1. I want to congratulate the authors for the effort and work done. It is a very important issue, given that, as mentioned, there is not enough information on infant mortality in low-middle-income countries, and there is not enough clarity on the association between acute diseases and mortality.
I consider you have done a good job, however I have some recommendations and corrections that I suggest

Thank you for this thoughtful review, we have addressed the comments point-by-point below.

2. Abstract: Add a little more background in the introduction to know the context and importance of this article

Please note that the background included in the abstract is limited by the recommended structured format, therefore we have not added further background.

3. Strengths and limitations: It is not clear what the limitations are, I suggest mentioning the possible biases that could have occurred, and what they did to avoid them

Thank you, the limitations have been added to this section as below. Further detail regarding potential bias and mitigation strategies are included in the discussion (page 23-24).

- **"This study was limited to the main district referral hospital in Sanghar district, Sindh therefore findings may not be generalizable to other areas in Pakistan, namely urban centers."**
- **"Only age, sex, diagnosis, and hospital disposition variables were available in the hospital registry data; therefore, it is not possible to characterize severity of illness, healthcare resource requirements, quality of care delivery, and their impact on hospital outcomes."**

4. I suggest checking the grammar in English

The grammar has been reviewed accordingly.

5. Methods:

The inclusion and exclusion criteria are not clear: were only those who were going to planned medical appointments excluded?

Only patients who were attending planned appointments (e.g., routine vaccinations, well-child visits) were excluded (Methods, page 9)

"Children presenting for planned elective visits were excluded (e.g., vaccinations, planned follow-up, routine well-child care, and elective surgery)."

The inclusion criteria were all children between 0-14 who consulted, including neonates, and patients with comorbidities?

Yes, every third consecutive patient 0 – 14 years with acute illness presenting to the outpatient department, emergency department and all patients 0 – 14 years admitted to the inpatient department were included (Methods, page 8-9). There were no exclusions for comorbidities.

"A structured case record form was used to extract data from the outpatient (including ED and OPD)..." I suggest adding the case record form as a supplementary file.

Thank you, a table containing the variables collected has been added to the supplementary file and referenced in text as below.

"A structured electronic case record form was used to extract data from the outpatient (including ED and OPD) and inpatient department registries daily throughout the enrolment period (Appendix 1). (Methods, page 9)"

-"Improbable entries (for example, neonatal diagnoses in children >28 days of age or age >15 years) were compared to the individual patient record and medical record as necessary and were marked as missing if incomplete." When participants were mentioned you described patients between 0 and 14 years old, you did not mention that neonates <28 days old were excluded. It is not clear if they were included or not.

Patients less than 28 days were not excluded. If a patient was not of neonatal age, however, and recorded as having a neonatal condition (for example, neonatal sepsis in a child > 28 days), their individual patient record was verified to determine whether there was an error in recorded age or diagnosis. Similarly, as patients > 15 years were not included in the study, the patient record of anyone in whom age > 15 years was indicated was verified to confirm their age and eligibility.

- I suggest adding the sample size calculation as a supplementary file

The sample size calculation was implemented using statistical software (R, Basic Functions for Power Analysis), referenced in-text.

6. Analysis:
I suggest that the methodology and analysis be reviewed by a statistician, since it is not my field of expertise.

Thank you. Dr. Bettina Hansen, a listed co-author, is a statistician who has reviewed the analyses.

You mention that the study was approved by the ethics review board. But you do not mention whether

any type of consent and assent was requested from the parents, guardians and/or patients participating in the study, which is important as it is a prospective cohort study.

Informed consent for participation was waived as data collection was limited to review of the patient registry and medical record. This has been edited in the methods section as detailed below.

"This study was approved by ethics review boards at Aga Khan University (2022-0979-21918), the Hospital for Sick Children (1000061181), and the University of Toronto (38710). Informed consent for participation was waived as data collection was limited to review of the patient registry and medical records and all data collected was de-identified. (Methods, page 10)"

7. Results and discussion:
I suggest explaining what diagnoses were considered communicable diseases and non-communicable diseases.

Thank you, a reference is included to indicate the categorization schema used, consistent with the Global Burden of Disease methodology.

"Recorded diagnoses were categorized according to sub-categories of the Global Burden of Disease 2017 (communicable diseases, non-communicable diseases, injuries, nutritional conditions, neonatal disorders, maternal conditions and ill-defined or cause unknown).(3)"

Of the patients who died at home, did they show clinical recovery upon discharge and subsequently became complicated? or since discharge they showed signs of complications?

Thank you for this important question. Three patients died at home among those in whom follow-up was complete. None of their families consented to participate in verbal autopsy and social autopsy, therefore details surrounding their end of life are unknown. One of these patients had been referred to another healthcare facility for further evaluation and management and two patients were discharged home. This has been added to the results a below.

"VASAs were not completed for the three children who died at home due to caregiver refusal; therefore no information regarding the circumstances of their deaths was available." (Results, page 17)

I consider reinforcing the discussion, for example, why do you think neonates were the most affected group, what are the risk factors for that age group?

The below has been added to reflect known risk factors for childhood mortality among neonates.

"Factors known to contribute to risk of neonatal death in Pakistan and globally include young maternal age, low maternal education level, socioeconomic disadvantage, short interpregnancy interval, inadequate antenatal care, place of delivery (e.g., facility versus home birth), prematurity, and small for gestational age birthweight."

I suggest making more comparisons with respect to the literature, not only from low-income countries, but also from developed countries.

The focus of this study is child mortality in low- and middle- income countries where the epidemiology of disease and burden of mortality differs significantly in high-income countries. Comparisons were drawn specifically to the Pakistan and South East Asia context as we expect this literature to be most relevant to the population studied.

I congratulate you for the effort made, it is a very important work, I consider it requires some corrections for its consideration to be published. I would like to see the corrections and the publication of the article in the future. Thank you for the valuable effort of contributing to the medical literature.

Reviewer: 2
Dr. Ibtihal Abdelgadir, Sidra Medical and Research Center, Doha
Comments to the Author:
Dear authors,

I would congratulate the authors for this good study,

Few points to be addressed and considered for improvements.

Abstract:
"this study found differences in presenting diagnosis based on age among children with acute illness seeking healthcare in Pakistan." This study is done in one hospital/center, with selective inclusions, so would not be reasonable to have that overall conclusion to include all healthcare in Pakistan.

Thank you, this sentence has been edited to reflect this as below.

"This study found differences in presenting diagnosis based on age among children with acute illness seeking healthcare in Sanghar district, Sindh, Pakistan."

Methodology
"We enrolled every third consecutive child aged 0–14 years presenting to the ED or OPD of the hospital and all children admitted to the inpatient department" I just wondered why not included all patients attended the ED or OPD, as that will get more robust results, especially when the results was stratified into few parameters including age and sex.

Thank you for this important point. The choice of sample size was guided by the power

calculation which indicated 2962 children required to detect a 10% difference in diagnosis frequency across 4 age categories and 7 diagnosis categories at a significance level of 0.05 and power of 0.80. We elected to enroll every third consecutive child as a feasibility measure to facilitate data collection in a high-volume, resource constrained setting.

VASA is done by trained personnel, please can you explain briefly the VASA and the "Three delay model," "then that was followed by cause of death assignment " explanation for the need of this step of assessment, as currently look like an extra step, that is not needed.

The following additional detail has been added to reflect the procedure of conducting a verbal and social autopsy (VASA) as well as the recommended standard for cause of death assignment following review of VASA transcripts.

"In the event of a death, a verbal and social autopsy (VASA) was conducted by trained study team members using the World Health Organization (WHO) 2016 verbal autopsy instrument and Institute for International Programs of the Johns Hopkins University social autopsy questionnaire.(15, 16) The VASA consists of an interview with the child's main caregiver regarding the child's medical history and the details of the illness that led to death. Additional questions are posed to understand social and behavioural determinants of death including household, community, and health system factors.(17) Cause of death is determined from the VASA by independent classification of VASA interview findings by a trained study physician. Cause of death was determined in our study independently and in duplicate by two physicians following a training set; disagreements were resolved by consensus or by a third reviewer (Sajid Soofi) as needed." (Methods, pages 9-10).

"A trained reviewer analyzed VASA transcripts using the Three Delays Model.(20) This framework characterizes causes of mortality related to barriers in the health-seeking process into three possible sources of delay including 1) delay in care seeking, 2) delay in reaching the healthcare facility, and 3) delay in appropriate treatment.(20, 21) By understanding of the potential sources of delay, interventions can be targeted to improve health outcomes." (Methods, page 11)

Data collection was done by accessing the patients' records, mainly retrospectively, can we clarify the prospective element of this study please

Patients were identified for inclusion prospectively and data were collected from the registry on a rolling basis over the enrollment period. This has been clarified in the methods as below.

"Based on feasibility considerations, we prospectively enrolled every third consecutive child aged 0–14 years presenting to the ED or OPD of the hospital and all children admitted to the inpatient department (IPD)."

Results
Data within the table might be better with some modifications as not easy to understand.

Please consider these comments for guidance.

Table 1

4 patients died in OPD, this is quite unusual as critically unwell patients, would be usually assessed in an emergency department, or to be shifted to a higher facility, unless death on arrival, please explain more.

Thank you for this comment. In many hospital settings, critically unwell patients would be triaged on arrival and seen in a resuscitation area of an emergency department and/or transferred to higher level of care. In this district headquarter hospital setting, pediatric patients may be seen in the outpatient department on a first-come, first-serve basis without undergoing triage. As a result, delay in assessment and management of critically unwell children may occur, contributing to risk of death in the OPD, or soon after admission to the IPD. None of the patients who died in the OPD were dead on arrival.

"Children were enrolled from the OPD as the majority of pediatric outpatients seek care for acute illness in the OPD on a first-come, first-serve basis, while the ED is predominantly frequented by adults. Children presenting to the ED, regardless of severity of illness, are routinely directed to the OPD for assessment and management." (Methods, page 8)

Table 2
The calculation of the number of patients who died, not very clear, two calculations were presented, for deposition and at 28 days. What is the cut off for both, and why not to add them together as overall mortality?

Thank you. The variable "disposition" has been clarified in the tables to indicate "hospital disposition". These recorded deaths are among patients who died during the index hospital admission. Cumulative mortality at 28 days has been corrected in Table 2 ("status at 28 days") to include all hospital deaths and all additional deaths identified in community follow-up.

"Of 1286 admitted children, 956 (74%) children were discharged alive, 229 (18%) were referred to another hospital, 58 (5%) left against medical advice, 43 (3%) died in hospital and, of 1096 for whom 28-day follow-up was complete, 3 (0.2%) died at home."

P values presented for all tables, assessed which variables, as there are multiple variables presented within the table, among different age groups and both sexes, please clarify?/

Thank you, we have noted in the footnotes that all comparisons are reported between age groups with aggregated sex.

"#p values are reported for comparisons between age group (aggregated sex)"

The conclusion about the mortality in children less than 28 days, associated trauma diagnosis can be discussed, however, the hospital under assessment on this study is not mentioned whether there is a neonatal unit/intensive care and/or the level of trauma service delivered. That would be part of the limitation of the study.

Thank you, this has been added to the description of the setting (Methods, page 8).

Kind regards,

Reviewer: 3
Dr. Adrian Esterman, University of South Australia
Comments to the Author:

Under the sample size section, please provide the effect size used.

Please see the below edit, reflecting the effect size of a 10% difference in disease frequency.

"For the primary analysis, 2962 patients were required to detect a 10% difference in diagnosis category distribution across 4 age categories and 7 diagnosis categories at a significance level of 0.05 and power of 0.80."

In the tables, please right adjust columns, and standardise the number of decimal points used.
Given the study design, why have you not used log binomial models rather than logistic regression models and obtained rate ratios rather than odds ratios?

Thank you, one decimal place is now used for all proportions unless ending with a trailing zero (not written). While a log binomial could be used for a dichotomous outcome, a limitation is that with few events the model may not converge. Therefore, we elected to use logistic regression and represent the results using odds ratios.

VERSION 2 – REVIEW

**REVIEWER**	Natalia Ante-Ardila<br>Hospital Universitario de la Fundación Santa Fe de Bogotá, Pediatrics
**REVIEW RETURNED**	11-May-2024

**GENERAL COMMENTS**	I congratulate you for the corrections made, and for the work done. It contributes to literature especially in developing countries such as Pakistan. It also provides information on the main pathologies in pediatric patients in rural areas, in such a way that it opens the possibility of proposing public health measures aimed at reducing them.<br><br>I suggest you make the following corrections:

	In the Abstract:<br>- “To describe presenting diagnoses and rates and causes of death by age category and sex among children presenting with acute illness to a district headquarter hospital in Pakistan”. The word presenting is repeated, I suggest changing it for a synonym<br>- I consider you can add some background to the abstract<br>- “The primary outcome was Global Burden of Disease diagnosis category. Secondary outcomes were 28-day mortality rate, cause of death, and health care delays”.<br>- What does "health care delays" refer to?<br>- I suggest reviewing the spelling and writing in English<br>- “Forty-seven children died in hospital” I suggest changing “in hospital” for “at the hospital”<br>In the introduction you could add what risk factors or determinants have been found to be related to the mortality of children between 5-14 years of age in Pakistan being 10 times higher than in developed countries.<br>What socio-cultural factors do you think are related to patients taking longer to seek medical care?<br>I congratulate you for including the population and epidemiology of the city where the study was carried out, which makes the context where the work was developed more understandable.<br>Very good that you added valuable information in the appendix section, which makes the study more reproducible.<br>Was the VASA interview carried out immediately after the patient died? Did you have any protocol to decide when to do the interview? Given that when the patient died, his caregivers were probably emotionally affected, and this could affect their responses in the interview. What actions did you take to prevent information bias in these cases?<br>I congratulate you for the comparisons you made in the discussion, on the results found, and those reported in the literature.<br><br>I consider the results found and the discussion carried out very valuable, I congratulate you again, I think you did a great job. I would like to see your work published

**REVIEWER**	Ibtihal Abdelgadir<br>Sidra Medical and Research Center, Doha , pediatrics
**REVIEW RETURNED**	18-May-2024

**GENERAL COMMENTS**	Dear authors,<br>Thank you very much, improved manuscript after implementations of the suggested changes. There are still a few grammar and language inaccuracies that needed to be addressed please.

**REVIEWER**	Adrian Esterman<br>University of South Australia, School of Nursing and Midwifery
**REVIEW RETURNED**	01-May-2024

**GENERAL COMMENTS**	Thank you for addressing those concerns.

VERSION 2 – AUTHOR RESPONSE

Reviewer: 3

Dr. Adrian Esterman, University of South Australia

Comments to the Author:

Thank you for addressing those concerns.

Reviewer: 1
Dr. Natalia Ante-Ardila, Hospital Universitario de la Fundación Santa Fe de Bogotá
Comments to the Author:

I congratulate you for the corrections made, and for the work done. It contributes to literature especially in developing countries such as Pakistan. It also provides information on the main pathologies in pediatric patients in rural areas, in such a way that it opens the possibility of proposing public health measures aimed at reducing them.

I suggest you make the following corrections:

In the Abstract:
- “To describe presenting diagnoses and rates and causes of death by age category and sex among children presenting with acute illness to a district headquarter hospital in Pakistan”. ◊ The word presenting is repeated, I suggest changing it for a synonym

> This has been changed as below.
>
> “To describe presenting diagnoses and rates and causes of death by age category and sex among children with acute illness brought to a district headquarter hospital in Pakistan”.

- I consider you can add some background to the abstract

> Please note that background is not included in the required structured abstract format; therefore, we have not acted on this suggestion.

- “The primary outcome was Global Burden of Disease diagnosis category. Secondary outcomes were 28-day mortality rate, cause of death, and health care delays”. What does "health care delays" refer to?

> Thank you; this has been edited as below.
>
> “The primary outcome was Global Burden of Disease diagnosis category. Secondary outcomes were 28-day mortality rate, cause of death, and health care delays, defined as delay in care-seeking, delay in reaching the healthcare facility and delay in appropriate treatment.”

- I suggest reviewing the spelling and writing in English

> Thank you; this has been completed.

- “Forty-seven children died in hospital” I suggest changing “in hospital” for “at the hospital”

> We have edited this sentence per below:
>
> “Forty-seven children died in the hospital.”

In the introduction you could add what risk factors or determinants have been found to be related to the mortality of children between 5-14 years of age in Pakistan being 10 times higher than in developed countries.

Thank you; we have added the following to the introduction:

> "Structural determinants of health that may contribute to higher mortality rates among certain populations of school-age children and adolescents in Pakistan include poverty, unsafe sanitation, food insecurity and nutritional deficiencies, air pollution, regional conflict, natural disasters, illiteracy, poor road traffic and transport infrastructure, and gender inequality."

What socio-cultural factors do you think are related to patients taking longer to seek medical care?

> Thank you for this question; the discussion (page 23) currently highlights certain factors that contribute to sources of delay in care-seeing, therefore no further edits have been made.
>
> "While caregivers may recognize signs of severe illness, the decision to seek care is often delayed for many reasons including cost, lack of transportation, distance to the facility, preference for home or traditional remedies, and lack of caregiver autonomy in decision-making.(43, 44) Delay in care-seeking contributes to increased severity of illness at the time of presentation to hospital, at which time the condition may no longer be reversible and this is compounded by treatment delay or delivery of poor quality care. Negative user experiences and perceived low quality of care at a given facility may contribute to future delays in the decision to seek formal care.(45) Moreover, caregivers may elect to bypass a facility altogether in favour of private health services or tertiary hospital care, potentially contributing to increase in transport time to reach definitive care.(45)"

I congratulate you for including the population and epidemiology of the city where the study was carried out, which makes the context where the work was developed more understandable. Very good that you added valuable information in the appendix section, which makes the study more reproducible.

Was the VASA interview carried out immediately after the patient died? Did you have any protocol to decide when to do the interview? Given that when the patient died, his caregivers were probably emotionally affected, and this could affect their responses in the interview. What actions did you take to prevent information bias in these cases?

> Thank you for this question. The VASA interview was conducted after the 28-day follow-up among all consenting caregivers of children who died. VASA interviews were conducted by research team members with experience using the method and prior training in conduct of VASA using the manual for the training of interviewers on the use of the 2022 WHO VA instrument and additional training tools from Johns Hopkins University Institute for International Programs. All interviews were conducted in the participant's primary spoken language. By using the standardized, chronologic tool, a comfortable interview process is ensured with emphasis on narrative components. Given that all VASAs were performed in the same manner and time by the same trained personnel, we would not expect systematic differences in data collection, recall, or analysis among participants. There is certainly potential for incomplete recall of events leading to the child's death; however, this possibility is mitigated by use of the structured, validated interview process. These considerations have been added to the limitations section of the discussion.
>
> "There is also potential for incomplete caregiver recall of events during VASA interviews which may contribute to under-reporting of sources of delay contributing to a child's death.

This limitation was mitigated by trained interviewers using a structured, validated process with an emphasis on eliciting a narrative, chronological summary of events."

I congratulate you for the comparisons you made in the discussion, on the results found, and those reported in the literature. I consider the results found and the discussion carried out very valuable, I congratulate you again, I think you did a great job. I would like to see your work published

Thank you for this thoughtful review.

Reviewer: 2
Dr. Ibtihal Abdelgadir, Sidra Medical and Research Center, Doha
Comments to the Author:
Dear authors,
Thank you very much, improved manuscript after implementations of the suggested changes. There are still a few grammar and language inaccuracies that needed to be addressed please.

Thank you; grammar and language have been reviewed accordingly.

VERSION 3 – REVIEW

**REVIEWER**	Natalia Ante-Ardila<br>Hospital Universitario de la Fundación Santa Fe de Bogotá, Pediatrics
**REVIEW RETURNED**	10-Jul-2024

**GENERAL COMMENTS**	I want to congratulate the authors for taking into account the suggestions that the other reviewers and I made. It is very pleasant to see the corrections you made, also the improvement in spelling and grammar in English is evident. I consider it, an interesting study, which I would like to see published. Congratulations again on the work and effort you put into making the improvements.
